# Set-level threshold-free tests on the intrinsic volumes of SPMs

**DOI:** 10.1016/j.neuroimage.2012.11.046

**Published:** 2013-03

**Authors:** Gareth R. Barnes, Gerard R. Ridgway, Guillaume Flandin, Mark Woolrich, Karl Friston

**Affiliations:** The Wellcome Trust Centre for Neuroimaging, UCL Institute of Neurology, Queen Square, London WC1N 3BG, UK

**Keywords:** Statistical parametric mapping, Random field theory, Intrinsic volume, Topological inference, Lipschitz–Killing curvatures, fMRI, MEG, TFCE

## Abstract

Conventionally, set-level inference on statistical parametric maps (SPMs) is based on the topological features of an excursion set above some threshold—for example, the number of clusters or Euler characteristic. The expected Euler characteristic—under the null hypothesis—can be predicted from an intrinsic measure or volume of the SPM, such as the resel counts or the Lipschitz–Killing curvatures (LKC). We propose a new approach that performs a null hypothesis omnibus test on an SPM, by testing whether its intrinsic volume (described by LKC coefficients) is different from the volume of the underlying residual fields: intuitively, whether the number of peaks in the statistical field (testing for signal) and the residual fields (noise) are consistent or not. Crucially, this new test requires no arbitrary feature-defining threshold but is nevertheless sensitive to distributed or spatially extended patterns. We show the similarities between our approach and conventional topological inference—in terms of false positive rate control and sensitivity to treatment effects—in two and three dimensional simulations. The test consistently improves on classical approaches for moderate (> 20) degrees of freedom. We also demonstrate the application to real data and illustrate the comparison of the expected and observed Euler characteristics over the complete threshold range.

## Highlights

► We calculate SPM intrinsic volume through the Euler Characteristic over thresholds. ► We compare these volume estimates to those obtained using classical methods. ► A multivariate test on these volume estimates gives a threshold free test of the null.

## Introduction

Random field theory is used in neuroimaging to account for the intrinsic smoothness of images, when making inferences on the basis of statistical parametric maps (Kilner and Friston, 2010; Worsley et al., 1996, 2004). Statistical parametric maps (SPMs) are realisations of random fields, whose topological characteristics—under the null hypothesis—can be predicted using random field theory. This provides a powerful framework for topological inference on data that are collected over one or more dimensions, such as images or time–frequency responses. In particular, random field theory allows one to make predictions about the Euler characteristic (EC) of the excursion set produced by thresholding a random field (intuitively, the number of blobs minus the number of holes of the excursion set). This prediction is useful, because—at high threshold—holes disappear and the expected EC approximates the number of maxima one would expect under the null hypothesis. This use of random field theory requires a threshold to create excursion sets that can then be assessed at various levels of inference (Friston et al., 1996); for example, on the basis of the extent or number of maxima above some threshold. In this work we introduce an approach that exploits random field theory without the need for a particular threshold.

The Euler characteristic of an SPM—at any threshold—can be predicted through the Gaussian Kinematic Formula (Taylor and Worsley, 2007). This formula expresses the expected EC as the sum over products of an EC density function and a measure of intrinsic volume. The EC density function depends only on the type of statistic that constitutes the SPM, and the intrinsic volume is a measure (curvature or count) of the multidimensional extent of the random field. In this work we use the *Lipschitz–Killing curvatures* (LKC) as our measure of intrinsic volume (Taylor and Worsley, 2007). However, it should be noted that LKC is interchangeable with the more traditionally used *resel count* through a scale factor.

**Fig. 1:**
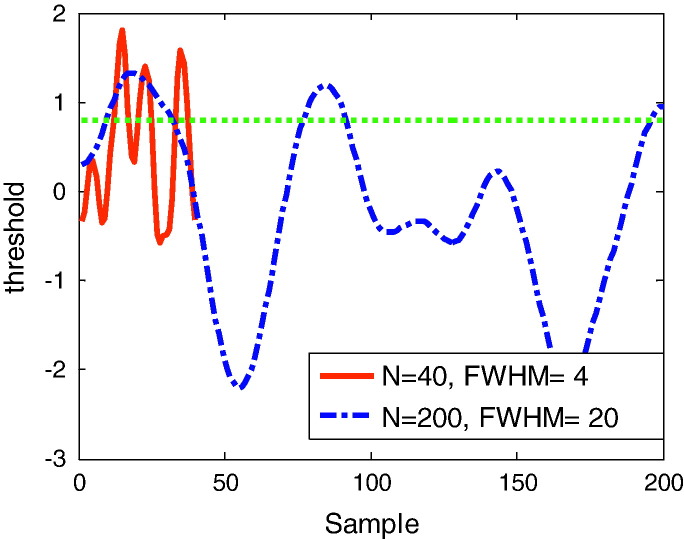
An example of two one-dimensional fields with identical intrinsic volume or Lipschitz–Killing curvature. Both curves are one-sample SPM{*t*}, based on 200 samples of Gaussian white noise. In one case (red—solid) the noise has smoothness FWHM = 4 and extends over 40 samples, in the other (blue—dotted) has FWHM = 20 and extends over 200 samples. The ‘intrinsic volumes’ of these curves, or the FWHM per unit length, are therefore identical (LKC = [1 16.65] or resels = [1 10]). The dotted green line is some arbitrary threshold *u* (= 0.8). The observed Euler characteristic for both processes at this threshold is 3 (there are 3 blobs above threshold). The Gaussian Kinematic Formula (Eq. (6)) for a random *t*-field of this intrinsic volume predicts an Euler characteristic of 2.9.

One can gain some intuition about the form of the Gaussian Kinematic Formula by considering a one dimensional statistical field or process (Fig. 1). For a given (high) threshold, the number of supra-threshold segments (the Euler characteristic) depends on the underlying statistic (determined by the density function) and a measure of the smoothness and length of the process (determined by the LKC). Smoother or shorter processes will have a smaller LKC and a smaller EC for a given threshold. Fig. 1 shows two *t*-statistical processes with the same intrinsic volume or LKCs: one is five times longer than the other but is also five times smoother. The intrinsic volume of a random field is the volume it would occupy when ‘statistically flattened’—so that it has unit smoothness everywhere. When flattened, the expected Euler characteristic is simply the EC density—per unit of intrinsic volume—times the intrinsic volume. In our one-dimensional example, both processes have the same intrinsic volume (the same underlying LKCs) and both are *t*-fields. Therefore, they have the same expected EC. In this case, the expected EC at this threshold (*u* = 0.8) is 2.9 and the number of observed supra-threshold segments is 3, in both cases.

These LKC or resel counts are usually determined from spatial gradients of standardised residual images over observations, such as trials or subjects (Kiebel et al., 1999; Kilner and Friston, 2010). This way of estimating the LKC implicitly accommodates any anisotropy in the smoothness of the original data (Worsley et al., 1999). Knowing the LKC allows one to compare the observed number of maxima above some threshold with that predicted under the null hypothesis and thereby compute a classical *p*-value (by appealing to the Poisson clumping heuristic; Friston et al., 1996). However, the threshold chosen can bias sensitivity to the detection of true effects in a way that depends upon the smoothness and amplitude of the true effects (Friston et al, 1996; Smith and Nichols, 2009). In what follows, we describe a procedure that eliminates the need to specify a threshold for omnibus or set-level inferences about SPMs. Although this level of inference does not apply to specific maxima within the SPM, it is generally the most sensitive inference available (Friston et al., 1996) and allows one to establish the significance of a treatment effect, which can then be localised using *post-hoc* tests.

Recent work (Bartz et al., in press) has exploited the fact that the Gaussian Kinematic Formula predicts the Euler characteristic, not just at high threshold levels, but at any threshold. This means that—rather than estimating the LKC from the smoothness of residual fields—one can simply solve a linear regression problem using the observed EC over a range of thresholds applied to a single residual field, or SPM.

In this work, we make use of the developments in Bartz et al. (in press) to estimate the LKC for every residual field (which are assumed to be realisations of Gaussian random fields) and for the final SPM (which—under the null hypothesis—is assumed to be a realisation of a central random field of *F* or *t* statistics). Under the null hypothesis these two measures of intrinsic volume should be the same, and so comparing these two sets of curvatures gives a direct and threshold free test of whether the final SPM deviates from the null hypothesis. In other words, instead of using the LKC to assess the significance of an excursion set (like the number of maxima above a threshold), we assess the significance of the LKC measure *per se*, and evaluate its null distribution using the residual images that have the same intrinsic volume but contain no treatment effect. Intuitively, we assess whether the numbers of peaks in the statistical field (testing for signal) and the residual fields (noise) are consistent or not. For example, if the SPM contains more maxima than it should under the null hypothesis it would appear to have a greater intrinsic volume.

The paper is divided into three sections. We first show the equivalence of the regression LKC estimators (Bartz et al., in press) and standard estimators used in statistical parametric mapping (Kiebel et al., 1999; Kilner and Friston, 2010). We then show that a simple multivariate test comparing the LKC of the residual fields and the SPM provides good control over false positive rates, and show that the method is comparable with techniques that rely on feature-defining thresholds. Finally, we demonstrate the approach on real fMRI data.

## Methods

We first review the basic mass-univariate GLM formulation that gives rise to the voxel-wise residuals. We then describe how these residuals are used to estimate a null EC distribution.

### Basic GLM

At each voxel the observations $y\in R^{N\times 1}$ over *N* subjects (or scans) can be modelled as a *p* column design matrix $X\in R^{N\times p}$ multiplied by a vector of regression coefficients $\beta \in R^{p\times 1}$ plus a vector of normal errors **e**.

(1) y=Xβ+e.

Ignoring relationships between elements of **y** over trials for clarity (see Discussion), the least squares estimator of the regression coefficients is

(2) $$\hat{\beta }={\left(X^{\text{T}}X\right)}^{-1}X^{\text{T}}y\text{.}$$

Leaving a vector of residuals $\hat{e}$ at each voxel

(3) $$\hat{e}=y-X\hat{\beta }$$

where the standardised residuals $r\in R^{N\times 1}$ are given by

(4) $$r=\sqrt{N-1}\frac{\hat{e}}{\sqrt{{\hat{e}}^{\text{T}}\hat{e}}}\text{.}$$

In the next section we will use the result that adjacent voxels will have similar standardised residuals.

### Random field theory

This section describes random field theory as typically used to quantify the number of maxima above a threshold one would expect by chance. This material is reviewed comprehensively in a number of other texts (Kiebel et al., 1999; Kilner and Friston, 2010; Worsley et al., 1996, 1999). For a *D* dimensional space *S*, let *A_u_* be the excursion set at threshold *u*.

(5) $$A_{u}=\left\{s\in S:A\left(s\right)\geq u\right\}\text{.}$$

For example, *S* might constitute a triangular mesh spanning the cortical surface with values for some statistical test at each vertex. Alternatively, it could be a regular lattice approximation to a three dimensional search space. *A_u_* would then constitute all the vertices above threshold *u*.

**Fig. 2:**
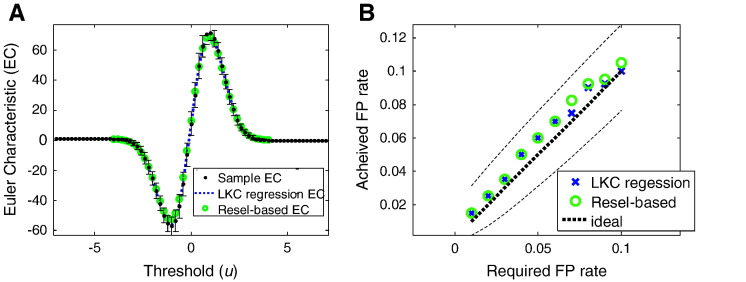
A. The average Euler characteristic as a function of threshold, for the standardised residual fields (black) with error bars showing the standard deviation over realisations. The green circles show the estimate of the Euler characteristic of the underlying random field—as predicted from the Gaussian Kinematic Formula (Eq. (6)) using the standard smoothness (resel) estimator (Eq. (9)). The blue dotted line shows the estimate of the Euler characteristic of the underlying random field as predicted from the average regression LKC estimates over realisations (Eq. (17)). B. The achieved false positive rate against the anticipated false positive rate controlling for a single supra-threshold cluster in the SPM. The threshold is set either by the standard (resel) estimator (green circles) or the regression estimator (blue crosses). This confirms that the curves in Fig. 2A are almost identical at high threshold. The dashed lines are binomial 95% confidence intervals around the anticipated false positive rate (black, dotted).

The expected value of the Euler characteristic of this excursion set (intuitively, the number of blobs minus the number of holes) is given by the Gaussian Kinematic Formula (Taylor and Worsley, 2007)

(6) $$E\left[\varphi \left(A_{u}\right)\right]=\sum_{d=0}^{D}L_{d}\left(S\right)\rho_{d}\left(u\right)$$

where *L_d_* are the Lipschitz–Killing curvatures (LKC) and depend on the smoothness and shape of *S*. Here, *φ*(*A*_*u*_) is the Euler characteristic of excursion set *A_u_* and *ρ*_*d*_ are EC density functions, determined purely by the statistic in question (usually a *t* or *F* statistic). *D* is the dimensionality of the image (e.g., 2 for a surface), where *D* = 0 corresponds to a single point. *L*_0_(*S*) is simply given by the Euler characteristic of the space under test (e.g. *L*_0_(*S*) = 2 for two separate hemispheric volumes; *L*_0_(*S*) = 4 for two separate surfaces with spherical topology). Having estimated the LKC for some space, one can infer (for example) whether two clusters (EC = 2) at threshold *u* = 3 would be expected by chance. Fig. 2 shows the correspondence between the empirical and predicted ECs from Eq. (6) using simulated data (see below).

If the space is not homogeneously smooth, it can be readily transformed by expressing distances (between vertices) in terms of the correlations between residuals (at each vertex) over observations (Worsley et al., 1999). This new space becomes uniformly smooth (statistically flat), where vertices with similar residuals are closer together. For a given dimension *d* ∈ {0,..,*D*}, each component (say triangle on a two dimensional mesh) will have *d* + 1 vertices. Now, indexing by component (*j*) let the *j*th component (say a triangle) have *d* + 1 vertices, each with a vector of standardised residuals $\left(r_{j_{0}},r_{j_{1}},\ldots ,r_{\mathrm{jd}}\right)$ (Eq. (4)). As we are only interested in the intrinsic volume occupied by this component (triangle), we can take one vertex (**r**_*j*0_) as a reference to define a *d* dimensional solid. Each component (triangle) is then defined by

(7) $$\Delta R_{j}=\left[r_{j_{1}}-r_{j_{0}},\ldots ,r_{j_{d}}-r_{j_{0}}\right]$$

where $\Delta R_{j}\in R^{N\times d}$. The volume (or area for a surface) of this *d* dimensional component is then simply given by

(8) $$Volume_{j}=\frac{1}{d!}{\left|\Delta {R_{j}}^{\text{T}}\Delta R_{j}\right|}^{1/2}\text{.}$$

For a triangle, this is half base multiplied by height. In the three dimensional case 8 adjacent voxel corners can be broken down into 5 tetrahedral components (Worsley et al., 1999). The Lipschitz–Killing curvature (Taylor and Worsley, 2007) of the whole space *L_d_*(*S*) is then simply the sum of the volumes occupied by the *J* individual components (triangles or tetrahedra).

(9) $$L_{d}\left(S\right)=\frac{1}{d!}\sum_{j=1}^{J}{\left|\Delta {R_{j}}^{\text{T}}\Delta R_{j}\right|}^{1/2}\text{.}$$

The equivalent resel counts are simply (4log(2))^−*d* / 2^*L_d_*(*S*). Finally, at high thresholds, when there are no holes in the image, the LKC given in Eq. (9) can be used in Eq. (6) to estimate the probability (or the expected value of the Euler characteristic) of a global maximum *M* of value greater than or equal to *u*

(10) $$P\left(M\geq u\right)\approx E\left[\varphi \left(A_{u}\right)\right]\text{.}$$

Extending the above to calculate the probability that the number of clusters (*c*_max_) should be greater than or equal to *C* clusters observed we can use the Poisson clumping heuristic (Friston et al., 1996)

(11) $$P\left(c_{\max}\geq C\right)=1-\sum_{c=0}^{C-1}\frac{e^{-\lambda }\lambda^{c}}{c!}$$

where the term on the right is the cumulative Poisson distribution with mean *λ* = *E*[*φ*(*A*_*u*_)], the number of clusters expected at threshold *u*.

When estimating the probability of a single cluster above threshold (*C* = 1), the approximation is consistent with Eq. (10) and *P*(*c*_max_ ≥ 1) ≃ *E*[*φ*(*A*_*u*_)].

There are two key points that arise in this use of the LKC. We have to specify a threshold to make use of the Gaussian Kinematic prediction (Eq. (6)). Second, in order to estimate the LKC, we need to know the local covariance structure of differences in residuals over observations and, implicitly, the topology or connectivity that defines these differences (Eq. (8)) (see Discussion).

### Estimating LKC through regression

In this section, we address the problem of estimating the intrinsic volume of a single realisation of a random field. This will allow us to decide whether the LKC of the SPM are consistent with those of the residual fields (in a group study there will be one per subject) that we know conform to the null hypothesis. By definition, the standardised residual at each vertex has a mean of zero and a variance of unity (Eq. (4)).

Each residual field corresponds to a *Z*-field, which has a predictable Euler characteristic over a range of *H* thresholds, *u_h_* (we used *H* = 81 thresholds from − 4 to + 4 with steps of 0.1). Following Bartz et al. (in press), we can then estimate the LKC using the following single general linear model:

(12) $$\varphi_{n,h}=\sum_{d=0}^{D}l_{n,d}\left(S\right)\rho_{d}^{z}\left(u_{h}\right)+\varepsilon_{n,h}$$

where *φ*_*n*,*h*_ is the measured EC at threshold *u_h_* of the *n*-th residual field (in a group study, *n* would index subjects). The unknown LKC of this residual field for dimension *d* is *l*_*n*,*d*_ and the superscript *z* in *ρ*_*d*_^*z*^(*u*_*h*_) signifies that this is the EC density for a Gaussian (rather than *t* or *F*) field at threshold *u_h_*.

At dimension zero, the LKC is simply the Euler Characteristic of the space under test and so this coefficient need not be estimated, giving

(13) $$\varphi_{n,h}-l_{n,0}\left(S\right)\rho_{0}^{z}\left(u_{h}\right)=\sum_{d=1}^{D}l_{n,d}\left(S\right)\rho_{d}^{z}\left(u_{h}\right)+\varepsilon_{n,h}$$

or

(14) $${\bar{\varphi }}_{n,h}=\sum_{d=1}^{D}l_{n,d}\left(S\right)\rho_{d}^{z}\left(u_{h}\right)+\varepsilon_{n,h}$$

where

(15) $${\bar{\varphi }}_{n,h}=\varphi_{n,h}-l_{n,0}\left(S\right)\rho_{0}^{z}\left(u_{h}\right)\text{.}$$

In order to simplify this notation, we can define the (data independent) *D* dimensional EC density (for a Gaussian) in Eq. (14) for all *H* threshold values as the matrix $\Gamma^{Z}\in R^{H\times D}$ and $l_{n}\in R^{D\times 1}$ as a vector of the (unknown) *D* Lipschitz–Killing curvatures (Eq. (9)) for residual field *n*.

We can re-write Eq. (14) in matrix form:

(16) $${\bar{\varphi }}_{n}=\Gamma^{Z}l_{n}+\varepsilon_{n}$$

where ${\bar{\varphi }}_{n}\in R^{H\times 1}$ is the matrix form of Eq. (15) measured over the *H* threshold values for residual field (subject) *n* and $\varepsilon_{n}\in R^{H\times 1}$ is a vector of errors.

Eq. (16) has a familiar form but there are some concerns about the assumptions required to solve this GLM. For example, the error terms are heteroscedastic (less variance at high thresholds) and the LKCs themselves are correlated (Bartz et al., in press). These authors examined a number of different covariance estimators (smoothed diagonal to account for heteroscedasticity, smoothed covariance to account for correlation, etc.) and found the simpler covariance models (ordinary least squares, smoothed diagonal) to be more robust (in terms of bias and variance).

Defaulting to the simplest regression model, here we estimate the LKC using ordinary least squares

(17) $${\hat{l}}_{n}={\left[\Gamma^{Z}\right]}^{+}{\bar{\varphi }}_{n}$$

where ${\left[\Gamma^{Z}\right]}^{+}\in R^{D\times H}$ is the pseudo-inverse of the Gaussian (*Z*) EC density and the estimate of the *D* unknown LKC coefficients of the *n*th (*n* = 1 to *N*) residual field is the vector ${\hat{l}}_{n}\in R^{D\times 1}$.

We can make a similar estimate of the LKCs underlying the test statistic image (which in the null case should be identical to those from the residual images)

(18) $${\hat{l}}_{\mathrm{test}}={\left[\Gamma^{t}\right]}^{+}{\bar{\varphi }}_{\mathrm{test}}$$

where ${\bar{\varphi }}_{\mathrm{test}}\in R^{H\times 1}$ has the same form as ${\bar{\varphi }}_{n}$ but is instead based on the measured EC of the SPM{*t*} over the threshold range. The LKC estimate for the test SPM is ${\hat{l}}_{\mathrm{test}}$. Note again that the superscript in **Γ**^*t*^ identifies this as the matrix of EC densities for the Student *t*-statistic.

We now can test whether the LKC estimated over the *N* residual fields are significantly different from those estimated from the SPM. Note that the use of the appropriate density function in Eq. (12) factors out any dependence on the statistic in question (*t* or *F*). We test for these differences using a standard multivariate general linear model:

(19) $$\left[\begin{array}{c} {{\hat{l}}^{T}}_{\mathrm{test}}\\ {{\hat{l}}^{T}}_{1}\\ \vdots \\ {{\hat{l}}^{T}}_{N} \end{array}\right]=\left[\begin{array}{cc} 1 & 0\\ 0 & 1\\ \vdots & \vdots \\ 0 & 1 \end{array}\right]\beta_{M}+E\text{.}$$

**Fig. 3:**
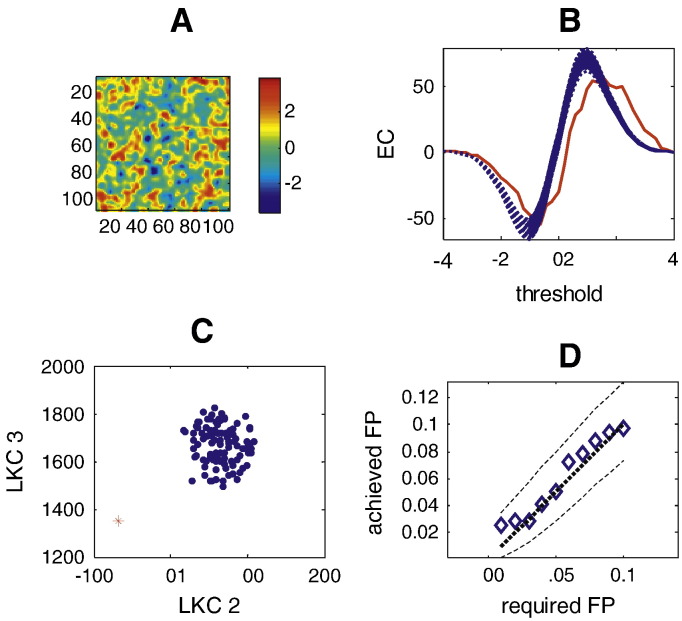
Panel A shows a one-sample SPM{*t*} (*N* = 100) on a 2D slice containing an underlying signal of 15 Gaussian blobs of FWHM 16 samples. Panel B shows the observed EC of the *t*-field (red solid) alongside the predicted EC of the *t*-field based on each of the LKC estimates for each of the 100 observations (blue dotted). Note that the EC observed does not conform to that expected; not just at high thresholds, but over the whole threshold range. The distribution of the per trial estimates of the 1st and 2nd dimension LKC estimates in B based on the residual fields (Eq. (17)) are shown in panel C as blue dots. The corresponding LKC estimates for the test image (Eq. (18)) are shown as a red star. It is clear that the test and the trial LKC estimates are unlikely to derive from the same distribution. Panel D shows the false positive rate of the multivariate test on the LKC coefficients for null data; dotted lines show ideal performance and the 95% confidence intervals.

This provides regression parameters, $\beta_{M}\in R^{2\times D}$ in which the first row is the LKC estimates for the test SPM, and the second row is the mean of the LKC estimates for the residual fields averaged over all samples or observations of the residuals. We then test the multivariate hypothesis that the two rows of $\beta_{M}\in R^{2\times D}$ are the same to provide a classical *p*-value. The test hinges on Wilks' lambda statistic, which is effectively a (marginal) likelihood ratio test comparing the full model to the reduced (null) model without the different estimates for residuals and test LKCs under Gaussian assumptions about the errors. For large *N* the log-likelihood ratio has a scaled Chi-squared distribution (Chatfield and Collins, 1980). In this case, we use Rao's F approximation to Wilks' lambda statistic (Anderson, 2003), to test whether the (intrinsic volume of the) SPM was sampled from the null distribution. The main assumption behind this test is that of multivariate normality. We used Mardia's test for multivariate normality based on the 3rd and 4th order moments of the distribution to test for this in the real data example (see Discussion); we also verified that the false positive rate (FPR) for this test was well controlled (Fig. 3D).

This concludes the description of our procedure that furnishes a simple test of the null hypothesis that the intrinsic volume of an SPM is the same as the intrinsic volume of its constituent residual fields—fields that contain no treatment effects. In the next section, we turn to numerical simulations to establish the accuracy and sensitivity of this scheme. All of this software is available from the authors on request and will be made part of SPM12.

## Simulations

We ran simulations using Gaussian white noise data for both two and three dimensional cases over volumes (or observations) of 100 × 100 pixels and (predominantly) 30 × 30 × 30 voxels respectively. For the two dimensional data, we used tests based on *N* = 100 observations, and for the three dimensional case we looked at *N* = 100, 50, 20 and 10 observations per test. We ran tests in batches of 400 random realisations to evaluate false positive rates (e.g., 400 tests with *N* = 100 random observations per test). To estimate receiver operating characteristic (ROC) curves, synthetic signals or treatment effects were created by adding a number (between 1 and 25) of impulse responses at random vertices (the same vertices across all slices/volumes). These signals were smoothed with a Gaussian kernel of varying width (FWHM varying from 2 to 32 voxels in steps of 2). The smoothness level (16 in total) and the number of signal peaks (25 in total) were varied systematically over realisations, giving 400 separate tests. To ensure that the fields were sufficiently smooth for random field theory, they were then smoothed with a Gaussian kernel of FWHM = 4. SPMs for each of the realisations were computed using one sample *t*-tests for a non-zero mean for each null and signal realisation. To compare our method to conventional procedures, we then used set-level tests with thresholds (but no cluster extent criteria) corresponding to uncorrected *p* values of 0.01 and 0.001 and a third test for any excursion above the RFT estimated *p* < 0.05 volume corrected threshold.

## Results

Fig. 2A shows the mean and standard deviation of the observed Euler characteristics (black) of the standardised residual fields over thresholds. Also shown are the Euler characteristics predicted from the Gaussian Kinematic Formula using the standard LKC estimates based on smoothness (Eq. (9)) and the LKC estimates based on regression (Eq. (17)). Usually, one uses the high threshold region of these curves to evaluate the probability of a maximum occurring by chance. Fig. 2B shows the false positive rate when testing for one or more clusters above threshold using the standard (local smoothness based) measure (Eq. (9)), alongside the false positive rate based on LKC estimates using regression (Eq. (17)). This confirms that the high threshold portions of the two curves in Fig. 2A are sufficiently similar to provide consistent false positive rate control.

By definition, adding signal to the data leaves the distribution of residuals unchanged (assuming the signal is modelled properly, see Discussion). However the resulting SPM will no longer be well described by the Gaussian Kinematic Formula—because it no longer conforms to a central statistical field under the null hypothesis. Fig. 3 shows an example of this. Panel A shows a one sample *t*-test on a realisation of 100 two dimensional fields containing an underlying signal. Panel B shows the observed EC for this test as a function of threshold (red solid). The expected behaviour of a random field SPM{*t*} based on LKC estimates from each of the 100 residual fields is overlaid (blue dotted). Note that the test EC is higher than would be expected at moderately high thresholds (*t* = 2 to 4), which is the basis of conventional set-level inference (based on the number of clusters above a threshold). However also note that the EC of this SPM is distinct from predictions based on the Gaussian Kinematic Formula across a wide threshold range. The distribution of these two estimated (*D* = 1, *D* = 2) LKC—based on the residual fields—is shown in panels 3C (blue dots) alongside the LKC estimates (red star) of this non-central SPM. This deviation of the trial and test LKCs is the basis of the multivariate test (Eq. (19)). In panel D we show that, over the 400 null realisations, the multivariate test has good control over the false positive rate.

**Fig. 4:**
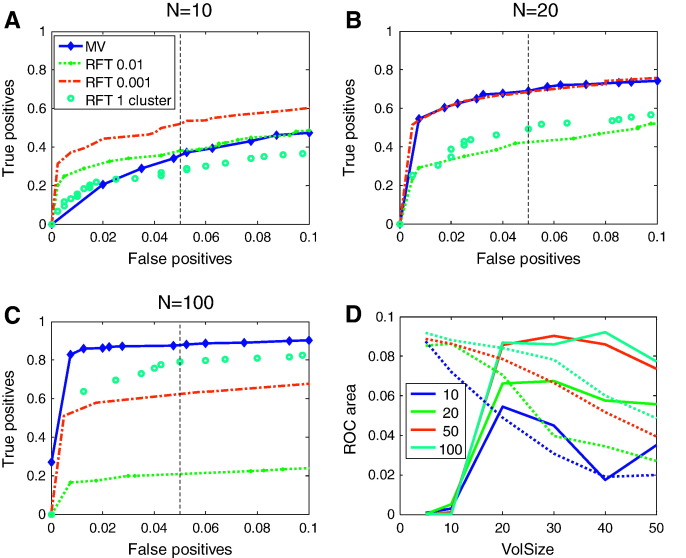
Panels A–C show receiver operator characteristic (ROC) for three typical set level tests: one or more clusters above *p* < 0.05 FWE corrected (cyan circles), number of clusters above a threshold corresponding to *p* < 0.01 uncorrected (green dots), number of clusters above *p* < 0.001 uncorrected (red dashed) and our multivariate test (blue diamonds). Panels A–C show the performance of these tests, in a volume of cube side 30, on SPM{*t*}s based on 10, 20 and 100 observations respectively. As the number of observations increases the multivariate test comes to outperform all the feature defined tests. Note that for 10 subjects, although the multivariate test is less sensitive than threshold-dependent tests, it has the fundamental advantage that it requires no search over thresholds (and corresponding statistical correction). Panel D shows the area under the ROC curve (false positive rate 0 to 0.1) against the side length of the cubic volume under test (x axis) for different numbers of observations (N = 10, 20, 50,100 in different colours) for the multivariate test (solid) and for (the classical) one or more clusters above *p* < 0.05 FWE corrected test (dotted). The performance in panels A, B and C correspond to the blue, green and cyan curves for a volume of side 30 voxels (FWHM = 4). The larger the number of observations the greater the potential improvement of the multivariate over the classical test, but for small volumes (*VolSize* < 20) the classical test (based on local smoothness) outperforms the multivariate test (based on the global EC count).

To evaluate the sensitivity and specificity of multivariate tests on the LKC we compared its performance with standard (based on local smoothness, Eq. (9)) set-level tests on the number of peaks above feature inducing thresholds corresponding to uncorrected *p*-values of 0.01 and 0.001 and a third test for one or more clusters above *p* < 0.05 FWE (Eq. (10)). We were also interested to find out how sensitive the approach would be for different subject numbers. Fig. 4 shows an ROC curve quantifying the performance of the multivariate test in comparison to the standard approaches. Panels A, B, C show the performance with *N* = 10, 20 and 100 observations respectively for volumes of side 30 voxels. The performance of the classical and multivariate approaches are similar for moderate *N* (= 20) with deterioration of the multivariate performance at low *N* (= 10). At *N* = 100 the multivariate test outperformed all chosen feature inducing thresholds for this volume. Note that although it is sometimes possible to find a feature-inducing threshold that outperforms the multivariate test (e.g. *N* = 20, feature threshold *p* < 0.001); in practice, searching for the best feature-defining threshold would incur a multiple comparison penalty (by inducing another dimension of the search space).

Panel D shows the area under the ROC curve (for false positive rates ≤ 0.1) as a function of both the number of observations (different lines) and image volume (x axis) for the (local smoothness based) classical test for one or more clusters above *p* < 0.05 FWE (dotted) and the multivariate method (solid). For small volumes or small numbers of trials the local smoothness based methods are more powerful; as the volume (or number of observations) increases, the EC estimates (over realisations) become less variable and the multivariate tests outperform the local smoothness based tests.

Existing parametric RFT relies on normality assumptions, and the new method is no different. In order to investigate dependence on Gaussian assumptions about error terms in more detail, we added an unmodelled (and un-physiological) step function to the simulated data of the same magnitude as the noise. The resulting non-Gaussian (bimodal) residual distribution was relatively benign for the smoothness based methods, resulting in a more conservative false positive rate; but for our method the effect was to give rise to capricious behaviour (see Supplemental Fig. S1A). Due to the step function, two dominant LKC clusters (each characterising half of the observations) emerged and their combination rather poorly described the EC of the final *t*-statistic field. We were able to address this effect, thanks to one of our reviewers, by calculating LKC estimates based on a rotated set of residuals (by multiplying the residuals from each of the *N* observations by the singular vectors of a random *N* × *N* matrix). This meant that the rotated (orthonormally mixed) residuals were not only more Gaussian (due to the central limit theorem) but also the variance due to this single step event was spread over the sequence of residuals, affording a better estimate of the true variance of the LKC coefficients (see Supplemental Figs. S2, S3). In the case of stationary data, rotation had no effect and could be applied to data where parametric assumptions about the error terms cannot be guaranteed (and the residuals can be exchanged among observations under i.i.d. assumptions).

**Fig. 5:**
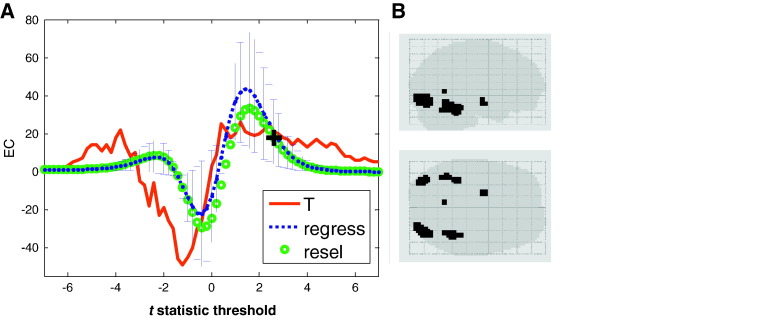
A. A one sample *t*-statistic (face–scrambled face) SPM comparison from an fMRI study with 18 subjects (Henson et al., 2011). The dotted blue line shows the predicted EC as a function of the *t*-statistic based on regression LKC estimates from the per-subject residuals. Error bars show the standard deviation from this prediction. The green circles show the predicted EC based on resel counts and image smoothness; note that these converge to the regression estimates at high threshold. The red solid line is the observed EC of the one sample *t*-statistic SPM. The LKCs of the test SPM differ significantly from those of the residuals (*F* = 8, *p* < 0.0019, df = [3,15]). The cross indicates the expected resel count at a feature defining threshold *p* < 0.01 (*t* = 2.57): at this point the predicted and observed EC are very similar hence the classical test (at this particular feature defining threshold) is not significant (*p* < 0.4). This highlights the disadvantage of the standard approach—in that only a single point of the observed (and high) EC is sampled. To sample other feature defining thresholds (e.g. *p* < 0.001) would incur a multiple comparison penalty. B. Given that we know the EC of the *t*-statistic is not due to random variation, we can now examine any portion of this curve (in a typical set level test we would only be able to examine the EC at the feature defining threshold). In this case, the supra-threshold clusters at *t* = 6 correspond to face specific areas.

Finally, we applied our method to an fMRI study with 18 participants (Henson et al., 2011; Wakeman and Henson, 2010). The experimental design involved the presentation of famous, non-famous and scrambled faces in random order. Here we simply look at the contrast between faces and scrambled faces. Fig. 5 shows the EC of the observed *t*-statistic over thresholds (red solid). Alongside is shown the predicted EC of a random *t*-statistic field based on the LKC estimates from the residuals using both smoothness based (green circles) and regression based (blue dotted) methods. Using the multivariate set-level test to compare the LKCs (as in Eq. (19)) we find that there is a significant effect (*F* = 8, df = [3 15], *p* < 0.0019). For these same data, the classical set level test (based on a feature defining threshold *p* < 0.01, *t* = 2.57) predicts an EC of 20 (black cross); as we happen to have unwarily selected a threshold at which the observed EC is also close to 20, the classical test is not significant (*p* < 0.4). If we now decide to test other feature defining thresholds using the classical approach we will have to account for multiple comparisons. Critically the multivariate test on the whole threshold range removes this problem. Panel B of Fig. 5 shows the exceedances at *t* = 6 (arrow in panel A) consistent with face processing areas.

## Discussion

In this work we have used the Gaussian Kinematic Formula to estimate the LKC of a single realisation of a random field. This allows us to estimate the LKC of an SPM assuming the null hypothesis and compare this estimate of its intrinsic volume to equivalent estimates of residual fields. In contrast to standard approaches this way of assessing the departure of the SPM from the null hypothesis, does not depend upon any threshold—rather it gives a complete characterisation of whether the Euler characteristic of the SPM, as a function of threshold, is consistent with the LKC estimated from component residual images. We note that other threshold free approaches exist—most notably permutation testing with threshold free cluster-enhancement (Smith and Nichols, 2009), which is however a non-parametric method. However, to our knowledge, this is the first parametric solution to this problem, and considers a complete characterisation of the field across all thresholds. One could consider this test to be the most general topological (set-level) inference; after which more specific *post-hoc* questions could be posed. The method has the same form irrespective of whether one wishes to make inferences on peak voxel intensity or some more diffuse global change in signal (Worsley et al., 1995). Notice that the procedures described in this paper can be applied to any SPM, including those based on serially correlated data (provided temporal correlations are modelled appropriately). This is because (standard) implementations of SPM use maximum likelihood estimators of effect sizes and therefore whiten the data (and residual fields) to render them approximately independent.

We note that the non-parametric TFCE (Smith and Nichols, 2009) procedure was developed with the same motivation in mind (to reduce the number of user specified parameters). Initial tests, show that the non-parametric TFCE also outperforms the individual feature defined tests with very similar performance to our parametric method (see Supplemental Fig. S4). The main strength of TFCE is that it is relatively immune to violations of Gaussian assumptions (see below); whereas the main advantage of our multivariate method (besides computational efficiency) is that there are no user defined parameters at all (no matter what the statistical test, as long as the associated random field has known EC densities). Our multivariate approach will suffer when the intrinsic volumes under test are small (see Fig. 4D) or the residuals are non-Gaussian and therefore there is some work needed to characterise the trade off between the non-parametric methods (such as TFCE) and our parametric multivariate method. Non-Gaussian residuals are generally not a problem in fMRI data due to the nature of image reconstruction and haemodynamic convolution, which render the data Gaussian by the central limit theorem. However, there could be circumstances (see Fig. S1) or other applications where departures from Gaussian behaviour may be more evident. Indeed multivariate methods may be particularly sensitive to violations of Gaussian assumptions and entail the additional assumption of multivariate normality. In the real data example, Mardia's test was not significant (*p* = 0.1863, 0.3016; corresponding to tests for multivariate skewness and kurtosis respectively). We also re-ran the real-data analysis using permutation testing (randomly labelling the tests and observations) and found a similar significant effect (*p* < 0.0205). This is clearly an area for future validation, with different data sets (VBM, MEG etc.), which can be easily verified through non-parametric methods.

One parameter—that could improve computational efficiency—is the step size and spacing of threshold levels over which to evaluate the empirical EC function. In this work, we used a fixed step size of 0.1 (*Z* or *t*), increasing linearly over the threshold range. The more threshold levels the better but this comes at some computational cost. This parameter was examined comprehensively by Bartz et al. (in press) who looked at the theoretical variance of the regression estimated LKCs as a function of step-size. They varied the number of threshold levels from 5 to 200 and found that the estimated variance plateaued at around 50. These authors also looked at different distributions of these levels (equal spacing, quantile spacing etc.). Ultimately these authors used 50 threshold levels over a linear range from − 3 to 3, which gives a comparable step size of 0.12. The authors used the same procedure to select a robust covariance estimator and ultimately selected a smoothed diagonal estimator, although they found that the ordinary least squares approach gave comparable performance. In this work we opted for the least complex model (the OLS) but the use of a smoothed diagonal estimator would be an interesting avenue for further work.

In this work, we make use of theoretical properties of a Gaussian random field to make a compact prediction of the LKC coefficients under the null hypothesis. An alternative, in the absence of expressions for the EC density of the test in question, might be to make an inference on the same residual and statistical fields (e.g. *Z*-fields) by comparing curve descriptions, using a method such as functional data analysis (Ramsay and Silverman, 1997) or summary measures of observation-wise estimates of extent and height similar to TFCE (Smith and Nichols, 2009). Indeed, initial tests using a simple univariate difference measure (between EC in test and EC in residuals over threshold) proved quite effective. The advantage of the parametric approach presented here is that the EC density allows us to predict the EC of any field (e.g. *F* or *t* fields) using standard topological theory.

As noted above this eschews an arbitrary feature defining threshold and therefore no search over thresholds (and implicit adjustment for multiple comparisons) are required for set level inference. Given that the set level test is significant one might then consider *post-hoc* tests with more localising power; for example, using standard tests on peaks or thresholded clusters. In this context, a significant effect at the set level provides protection for localised tests. In other words, having established a significant effect using threshold-free inference, one can then report local tests without further correction for multiple post-hoc tests. This is because the false positive rate of *post-hoc* (localised) tests can never exceed the nominal false positive rate, provided one does not proceed to *post-hoc* testing in the absence of a significant test at the set level. Clearly, false positive rates for local tests are only controlled in a (technically) weak sense. On this note, some localising power is possible with the algorithm if a specific anatomical region is specified *a priori*. From Fig. 4D it is clear that the main advantages of using the multivariate framework become apparent at intrinsic volumes of around 125 resels (20 voxel cube side at 4 mm smoothing).

The ability to estimate the LKC coefficients of a single statistical field allows one to test for signal-induced changes in the apparent intrinsic volume (as estimated through regression). For example, individuals with more extensive horizontal connections in V1 (Schwarzkopf et al., 2012) might give rise to a quantitatively distinct noise field. The LKC coefficients may be a principled and compact parameterisation of the spatial correlations in neuronal fluctuations that could be used to test for such effects.

As pointed out by Bartz and colleagues, the regression method provides a computationally and conceptually simple LKC estimator. It avoids the rather complicated calculation of neighbourhoods (Worsley et al., 1996) and, importantly, is easily generalised to any number of dimensions. As no geometric knowledge of the field is necessary (but simply its topology), exactly the same methodology can be applied directly to tests on two dimensional cortical surfaces or high dimensional connectivity images (by simply changing *D* in Eq. (6)). For example, in MEG, source orientation as well as Euclidean distance determines the covariance between voxels (Barnes et al., 2011) and the true topology is not necessarily that of nearest neighbours. The use of persistent homology (Adams and Carlsson, 2009) allows one to estimate the topology—and Euler characteristic—of sets of arbitrary data points. This means that one can exploit the simplicity of the regression approach to estimate the LKC of fields with unknown dimension or topology. We will explore this important application in future work on topological inference in MEG data that can show a complicated correlation structure and statistical topology.
