# Supplementary material for: Set-level threshold-free tests on the intrinsic volumes of SPMs
Source: Neuroimage. 2013 Mar;68:133–40. doi: 10.1016/j.neuroimage.2012.11.046 (PMC3625125; doi:10.1016/j.neuroimage.2012.11.046)
Supplement: Supplementary file 1 — Supplementary materials. [file mmc1.doc]

**Supplementary information**

**Figure S1.**

The effect of unmodelled step function, giving rise to non-Gaussian residuals, on the multivariate test. Panel **A** shows that this causes a dramatic increase in the false positive rate of the multivariate test (blue diamonds) driving it to be extremely capricious (compare the dotted lines showing ideal performance). Whereas smoothness based statistics (green circles) became moderately conservative (failing gracefully). Panel **B** shows the deviation between the expected EC based on the residual fields (blue curves) and the test statistic (which should be within this distribution in the null case). Panel **C**, **D** show that his behaviour is due to two distinct sets of LKC coefficients fitting the data (right lower panels) corresponding to the two halves of the step function. This bimodal data is clearly not normal and our inference becomes anti-conservative.

**Figure S2.**

The same data as in Figure S1, but a random NxN rotation matrix to create new (rotated) per trial residuals (for the multivariate test only). Panel **A**shows how the performance of the multivariate test (blue diamonds) becomes conservative and comparable to that of the (unrotated) local smoothness result (green circles). Panel **B** shows that the EC of the test statistic now conforms with those of the residuals. Panels **C,D** show that the rotation has the effect of spreading the effect of the step function over all realisations, making the final distribution more Gaussian (less bimodal) and hence improving our inference.

**Figure S3.**

This figure is based on the same set up as Figure 4, but with the addition of an unmodelled step function (equal to the standard error of the noise) occurring halfway through the observations. The classical, local smoothness based approaches still perform reasonably well in these conditions (RFT symbols). However the manipulation completely undermines the multivariate method (blue diamonds). Using the rotated residuals however (green squares) provides relatively graceful degradation of the multivariate test.

**Figure S4.**

The same simulation as in figure 4C (100 observations, cube side 30 voxels), but including non-parametric maximum *t* statistics (purple triangles) and the non-parametric TFCE (with extent and height parameters set to 0.5 and 2 respectively) measure (yellow circles). As expected the parametric and non-parametric statistics based on the global maximum (cyan circles and purple triangles respectively) have similar behaviour. Note that the performance of TFCE (yellow squares) is very close to that of the parametric multivariate method we propose (blue diamonds).
